# Breaking point: the genesis and impact of structural variation in tumours

**DOI:** 10.12688/f1000research.16079.1

**Published:** 2018-11-19

**Authors:** Ailith Ewing, Colin Semple

**Affiliations:** 1 MRC Human Genetics Unit, MRC Institute of Genetics and Molecular Medicine, The University of Edinburgh, Western General Hospital, Crewe Road, Edinburgh, EH42XU, UK

**Keywords:** Structural variation, cancer, tumourigenesis, DNA double-strand breaks, mutational signatures, whole genome sequencing

## Abstract

Somatic structural variants undoubtedly play important roles in driving tumourigenesis. This is evident despite the substantial technical challenges that remain in accurately detecting structural variants and their breakpoints in tumours and in spite of our incomplete understanding of the impact of structural variants on cellular function. Developments in these areas of research contribute to the ongoing discovery of structural variation with a clear impact on the evolution of the tumour and on the clinical importance to the patient. Recent large whole genome sequencing studies have reinforced our impression of each tumour as a unique combination of mutations but paradoxically have also discovered similar genome-wide patterns of single-nucleotide and structural variation between tumours. Statistical methods have been developed to deconvolute mutation patterns, or signatures, that recur across samples, providing information about the mutagens and repair processes that may be active in a given tumour. These signatures can guide treatment by, for example, highlighting vulnerabilities in a particular tumour to a particular chemotherapy. Thus, although the complete reconstruction of the full evolutionary trajectory of a tumour genome remains currently out of reach, valuable data are already emerging to improve the treatment of cancer.

## Introduction

Tumours arise when normal cells accumulate enough genetic alterations to affect normal regulatory control systems ^1^. These alterations usually include somatic structural variants (SVs) which are large-scale changes to the genome, ranging from 50 nucleotides to many megabases in length ^2^. SVs can occur as a result of improper DNA double-strand break (DSB) repair ^3^ and are traditionally classified into five different types: insertions, deletions, duplications, inversions, and translocations ^2^. Although all tumours accumulate both somatic single-nucleotide variant (SNV) mutations and SVs, certain tumour types such as high-grade serous ovarian cancer (HGSOC) and invasive breast cancer ^4^ are dominated by SVs. Furthermore, in HGSOC ^5^ and pancreatic neuroendocrine tumours ^6^, there is good evidence for SVs playing prominent roles in driving tumourigenesis. Various sequencing strategies, particularly whole genome sequencing (WGS), can be used to identify structural variation in tumours by comparing the pattern of structural variation in tumour tissue with that observed in normal cells ^2, 7^. A range of computational tools are then used to detect these variants from sequencing data, but detection algorithms perform very differently in identifying particular SV classes, and none of the existing tools are effective in detecting all SVs on their own ^8^. Copy number alteration (CNA) detection in cancer samples has been reviewed extensively ^9– 11^ because of its important role in tumourigenesis ^12, 13^. For example, it has been suggested that HLA allele loss in 40% of non-small cell lung cancers enables immune escape ^14^. The detection of CNA is a more tractable problem than the detection of many other SVs, as CNAs can be inferred purely on the basis of changes in read depth over a region. Regions with lower read depth suggest loss of genetic material, whereas regions of higher read depth suggest gain. Aside from detecting copy number changes, analyses of SVs in the past have focused on investigating simpler rearrangements of known cancer genes or identifying fusion genes ^15– 19^; however, the focus is now shifting to considering more complex events and SVs as part of broader, genome-wide patterns of mutation ^20^. Our present knowledge of structural aberrations is clearly far from complete, such that although aneuploidy is well studied ^21– 23^, large-scale phenomena such as whole genome doubling have only recently been discovered to be a common event in many cancers and to have important implications for clinical prognoses ^24^.

Even when an SV is associated with patient survival, it is challenging to uncover the underlying mechanism (that is, how the SV impacts tumour function). It is known that SVs can alter the expression of oncogenes or tumour suppressor genes and that these changes in expression may directly contribute to tumourigenesis ^3, 8, 25– 27^. It also seems that SVs with breakpoints in the main body of a gene may often be associated with increased expression of the gene but that SVs with breakpoints within 50 kb upstream or downstream of a gene are correlated with decreased expression ^25^. Similarly, it has been observed that amplified regions of the genome are enriched for oncogenes but that deleted regions are enriched for tumour suppressors ^1^. However, SVs may alter the expression of genes through various mechanisms, and multiple SVs may occur at the same locus, so the impact of a particular SV on nearby genes is often difficult to predict ^25^. SVs have long been known to result in the formation of functional fusion genes by translocations ^1^. For example, the BCR1-ABL fusion gene is formed by a t(9;22)(q34;q11) translocation which leads to increased tyrosine kinase activity, uncontrolled proliferation, and ultimately chronic myeloid leukaemia (CML). This type of gene fusion can be targeted with kinase inhibitors, resulting in an effective treatment for CML. Rearrangements can also reposition regulatory elements such as enhancers next to oncogenes, which has been observed in Burkitt’s lymphoma and results in increased expression of the *MYC* oncogene ^1, 25^. Recent studies have suggested that enhancer adoption by oncogenes following the disruption of regulatory domains is fairly common in tumourigenesis, occurring at rates comparable to those of recurrent in-frame gene fusions ^28^, although the domain architectures themselves may simply be prone to higher rates of mutation ^29^. Finally, there appear to be SVs with breakpoints that do not have direct effects on either genes or regulatory elements and yet their presence is associated with patient survival rates ^1^. Beyond gene expression, the effects of SVs are not well studied, but notably modest correlations have been reported between the extent of tumour duplications and deletions involving a gene and the levels of the protein it encodes ^30^. In summary, although simpler SVs may tend to have intuitive effects on gene expression (for example, deletions leading to lower expression and amplifications leading to higher expression), it is generally not straightforward to interpret the functional effects of a given SV within a given tumour.

## Identifying structural variation

Historically, chromosomal aberrations were detected by using microscopy and traditional karyotyping methods, which then were improved with the development of fluorescent *in situ* hybridisation techniques ^31^. Modern high-throughput sequencing technologies are capable of detecting a wider variety of structural variation at much higher resolution. The most widely used sequencing technologies generate billions of short reads about 100 nucleotides in length from sequencing libraries ^8^. The short lengths of the reads, sequencing platform error rates, and the sequencing libraries used can all present challenges for SV detection. Some reads map ambiguously to many different regions in the reference genome, which makes it difficult to determine the true location of the corresponding SV. This is particularly true of reads in repetitive regions ^8^. Smaller library insert sizes enable the precise identification of small SVs such as insertions and deletions; however, smaller insert sizes will cover only short stretches of sequence, making it hard to cover large SVs ^2^. Contrastingly, larger insert sizes allow the identification of large SVs but may have insufficient coverage to determine the location of breakpoints at base-pair resolution. Given the high cost of conducting sequencing experiments using multiple library sizes, it is important to use the appropriate strategy for the biological hypothesis under study. Chimeric reads are artificial sequences formed during the amplification step of polymerase chain reaction (PCR) and can also result in artefacts during analysis. The effect of chimeric reads on SV prediction can be mitigated by using multiple libraries per sample and filtering out novel sequences that are in only one library; however, this comes with the risk of filtering out low-frequency SVs ^2^. Alternatively, there are tools such as ChimeraChecker ^32^ and ChimeraSlayer ^33^ which are designed to remove chimeric reads. Finally, the genomic context of the sequence also impacts SV calling. Regions that are poorly characterised in the reference genome such as pericentromeric and telomeric regions present a challenge to the identification of SVs. In addition, repetitive or GC-rich regions are often problematic for confident read mapping, which can be overcome by sequencing technologies generating longer reads ^2^.

Several long-range technologies have been developed to try to address these challenges ^34^. PacBio ^35^ and Oxford Nanopore ^36^ sequencing platforms both produce long reads that can be used for SV detection. The resultant reads from these technologies average about 10 kb in length and some read lengths recorded up to 100 kb for PacBio and 1 Mb for Oxford Nanopore. However, these technologies are more expensive than standard Illumina short-read sequencing and have higher error rates. An alternative approach is to use a technology based on linked short reads, such as the platforms developed by 10X Genomics ^37^. This enables the identification of large SVs at a lower cost than for the long-read technologies; however, sequencing coverage can be relatively sparse, limiting the resolution of locally repetitive sequences ^34^. Although they are established, these technologies are currently not widely used in cancer genomics and the majority of SV detection in tumours for the moment is still carried out on the basis of short-read sequencing data.

Most SV detection algorithms or “callers” identify SVs from paired-end sequencing reads that are anomalously mapped. Anomalous reads can be used to predict candidate SV loci because reads that do not contain an SV breakpoint are expected to map concordantly ^2^. Anomalously mapped reads fall into one of three categories: discordant reads, soft-clipped reads, and one-end-anchored reads. Discordant reads can be used in the direct discovery of SVs and are mapped to either different chromosomes (providing evidence for translocations) or incorrect strands (suggesting inversions) or are in incorrect orientation (suggesting duplications) or are an incorrect distance apart (indicating the presence of an insertion or deletion). Detecting SVs from discordant reads is more effective for the detection of larger SV events, as they are unable to estimate breakpoint locations precisely and small SVs may have insufficient discordant read coverage to be detectable ^8^. Soft-clipped and one-end-anchored reads can be used for indirect SV discovery. Soft-clipped reads are only partially mapped, and one-end-anchored reads have only one read of the pair mapped. In both cases, split-read mapping is needed to successfully map both ends of the SV. These categories of anomaly are better for detecting smaller events such as small insertions and deletions, as they are able to estimate the breakpoints at a higher resolution.

In practice, SV callers use one or more types of anomalous reads to predict candidate SV loci in the following ways ^2^: first, by clustering discordant reads that support the same type of SV together (for example, SVDetect ^38^); second, by using split-read mapping to map the other end of indirectly discovered SVs (for example, BreakSeq ^39^) or to refine the estimate of the breakpoint location in directly discovered SVs (for example, LUMPY ^40^); and, third, by using *de novo* contig assembly to stitch together short reads to make a longer segment that can be mapped back to the reference and improve the ability to locate breakpoints (for example, CREST ^41^). Some callers use combinations of all of these techniques, in addition to assessing changes in read depth, in order to improve their detection sensitivity (for example, DELLY ^42^).

Assessing the accuracy of SV callers has proven to be challenging. The International Cancer Genome Consortium–The Cancer Genome Atlas (ICGC-TCGA) DREAM SMC-DNA Challenge ( https://www.synapse.org/#!Synapse:syn312572/wiki/) was launched in 2013, aiming to benchmark somatic SNV and SV predictions from a wide variety of algorithms using simulated and real tumour-normal WGS pairs. The project revealed widespread biases in caller predictions and recommended the use of consensus calls made by multiple callers for both SNV and SV prediction ^43^. More detailed accounts of the SV callers’ performance showed that several algorithms (including Breakdancer, DELLY, Pindel, Manta, and novoBreak) could achieve high precision after stringent filtering of SV calls and substantial overlaps in the breakpoints called between them ^44^. Unfortunately, SV calling is a rapidly evolving field, and there is no ongoing attempt to comprehensively benchmark algorithms as they appear or are updated. In spite of this, multiple comparative reviews of the most popular SV detection tools and algorithms have emerged ^8, 45, 46^. The ensemble approach to both SNV and SV calling explored in the DREAM Challenge has since become the *de facto* standard operating procedure in the field, as reflected in the recent re-analyses of large TCGA ^47^ and ICGC ^48^ tumour sequencing studies. The choice of algorithms employed in such an ensemble is often influenced by practical considerations, such as computational demands and run times, but should also consider the shared assumptions and biases between tools ^49^. Because any ensemble is expected to generate false-positive and false-negative results, there is also a requirement for validation by orthogonal data for SVs of particular interest, and long-read sequencing technologies may often be useful in this respect ^50^.

## Genesis and patterns of structural variation

SVs arise when DNA DSBs are improperly repaired ^51^. The three main DSB repair mechanisms are homologous recombination (HR), non-homologous end joining (NHEJ), and replication-based mechanisms ^3, 52^. HR is the most common DNA repair mechanism ^3^ and is accurate as long as there is a homologous sequence to use as a repair template. However, in tumour genomes, there are few overlapping sequences at the breakpoints ^53, 54^, so generally HR is not possible and the error-prone NHEJ is implemented instead. NHEJ requires no homology and sometimes can generate short segments of microhomology or small insertions at the breakpoints ^3^. Replication-based mechanisms of DNA DSB repair such as microhomology-mediated break-induced repair (MMBIR) have also been described ^55^. It is thought that in these cases the replication fork can stall and the polymerase can switch template sequence using microhomology to any nearby single-stranded DNA ^3^. This can result in a range of complex structural rearrangements ^56, 57^. In tumour genomes, which are often HR deficient, these last two mechanisms of repair dominate ^3^. A further mechanism behind structural variation in cancer is chromosome instability. This is defined as an increased rate of chromosomal change in comparison with normal cells. Nearly all cancer cells have some degree of chromosomal instability, but it is unclear whether this drives tumourigenesis or is a by-product of cancer evolution ^58^. There is evidence to suggest that a background of chromosomal instability accelerates the rate of tumourigenesis aided by the acquisition of mutations that promote tolerance of instability (for example, inactivation of *TP53*) ^58^. Furthermore, the role of recombination activating gene (RAG) and activation-induced cytidine deaminase (AID) in the diversification of immunoglobulin-encoding genes has been proposed as a driver of SVs in leukaemia. The RAG1 and RAG2 endonucleases introduce DSBs followed by the recombination of the immunoglobulin variable, diversity, and joining regions. AID deaminates cytosine residues in the immunoglobulin switch and variable regions, allowing somatic hypermutation. This genetic diversification of antibodies is activated in response to infectious or inflammatory stimuli and has been shown to contribute to the accumulation of somatic mutations necessary for clonal evolution in leukaemia ^59, 60^. The mechanisms underlying the generation of structural variation outlined above have been reviewed extensively elsewhere ^1, 3, 51, 61, 62^.

The traditional model of cancer development involves the gradual sequential accumulation of mutations, which leads to gradual progression of normal tissue through increasingly disordered clinical and pathological stages until it becomes a malignant tumour ^54^. An alternative model was suggested by Stephens *et al*. to explain complex patterns of genomic rearrangement ^54^. They suggest a catastrophe-like model where multiple SVs occur as a result of a single event ^54^.

It is likely that such a model of mutation is behind complex patterns of genomic rearrangements found in cancer, such as chromothripsis and chromoplexy ^54, 63– 66^. Chromothripsis is characterised by tens to hundreds of genomic rearrangements across an extended chromosomal region. A region that has undergone chromothripsis shows the following hallmarks: oscillating copy number between usually two copy number states; the retention of heterozygosity in the regions with the higher copy number state; and clustering of SV breakpoints to a greater extent than would be expected given a background model of the regional propensity to rearrangement ^54^. Chromothripsis appears to be present in 2% to 3% of all cancers and has a higher prevalence in bone cancers (25%) ^54^. It has been reported to be associated with the presence of *TP53* mutations in tumours of diverse origins, including medulloblastomas, acute myeloid leukaemia, and pancreatic tumours ^67– 73^, suggesting a connection to general genome instability. Moreover, chromothripsis has been associated with poorer prognosis in neuroblastoma ^74^, acute myeloid leukaemia ^70, 73^, multiple myeloma ^75^, and malignant melanoma ^76^. In contrast, it has been linked with longer progression-free survival in colorectal cancer ^77^. This suggests that chromothripsis has the potential to be an informative biomarker in tumours but with implications that may differ by cancer type. A further complex pattern of structural rearrangements is chromoplexy ^78– 80^. This is characterised by chains of translocations and deletions that are interdependent. It differs from chromothripsis in that it involves fewer rearrangements per chain (3–40+) and the rearrangements can be spread across up to 10 chromosomes. It is thought that multiple chains of chromoplexy can be formed in successive cell cycles but that all of the SVs in each chain are likely to be formed by one event ^63^. A further sign of chromoplexy is the presence of small deletions between fusion junctions of some translocation rearrangements known as “deletion bridges” ^63, 79^. Chromoplexy is largely copy number neutral and has a high prevalence in prostate cancers (88%) ^63, 78, 80^.

Rates of structural mutation vary across the genome, and there are some regions of the genome that are more susceptible to recurrent structural mutation than others. Glodzik *et al*. ^81^ used a large WGS breast cancer cohort ^20^ to identify such regions that appear to harbour greater recurrent structural variation than expected given the genomic characteristics of the region. These characteristics included replication timing domains, gene-rich regions, background copy number, chromatin states, and repetitive sequences. Many of the rearrangement hotspots found were dominated by long (greater than 100 kb) tandem duplications. These hotspots often duplicated key driver genes and regulatory elements and were enriched for breast cancer susceptibility loci and tissue-specific super enhancers. Thus, based upon the functional regions included, some fraction of these hotspots may represent driver events under selection in tumours, but many hotspots may also be generated by mutational bias at these regions (such that they are more prone to mutation or less accessible to repair) mediated by their chromatin structure and replication timing ^81^. Regardless of the relative roles of selection or other processes in their origins, as we discuss below, these patterns of recurrent structural mutation may be clinically informative.

## From sequence to data to treatment

**Figure 1.**
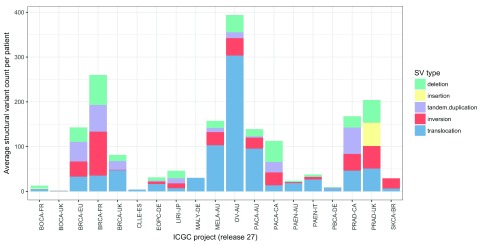
Average number of structural variants per patient by cancer type. The average number of structural variants per patient varies across 19 whole genome sequencing studies of cancer from the International Cancer Genomics Consortium (ICGC) (release 27). Structural variants were classified into five types: deletions, insertions, tandem duplications, inversions, and translocations. Some tumours such as ovarian, breast, and prostate have relatively large numbers of structural variants, whereas bone cancers and chronic lymphocytic leukaemias have relatively few. However, biological differences among tumour types can be confounded by technical differences among studies. SV, structural variant.

The role of genomics in precision therapy often focuses on using targeted sequencing technologies to test for the presence of clinically relevant SNVs. However, certain tumour types are characterised by modest SNV loads and instead show extensive structural rearrangements ^4, 82, 83^. Indeed, Ciriello *et al*. ^82^ suggest that there is an inverse relationship between the burden of SNVs and CNAs across tumour types (Figure 2a of 82). Figure 1 illustrates the average number of SVs per patient observed across 19 projects from ICGC ^84^ (release 27). Although it can be challenging to adjust for the inter-study differences highlighted below, it appears that certain (e.g. ovarian, breast, and prostate) tumours possess high frequencies of SVs but that other tumour types such as bone cancers and chronic lymphocytic leukaemia do not. However, there are also noticeable differences in the frequencies of mutations reported between cohorts of the same tumour type, which may reflect differences in cellularity, sequencing depth, and the SV callers used across studies. These differences emphasise the challenges that exist in measuring the degree of structural variation across studies with multiple confounding factors. Nevertheless, the clinical utility of certain SVs is already established. Currently, clinically actionable SVs include gene fusions ^85^ (for example, BCR-ABL), oncogene amplifications (for example, *ERBB2*), and tumour suppressor deletions (for example, *BRCA1*). Mertens *et al*. ^85^ discuss the impact of gene fusions in cancer and describe their utility as effective drug targets (Table 2 of 85). Further advances in technology will be necessary for the reliable identification of more complex SV patterns in a clinical setting where low-cost and rapid-turnaround assays are desirable ^83^.

In the past, the analysis of somatic mutations in cancer has often focused on recurrent driver mutations located within the small fraction of the genome encoding proteins and thought to confer selective advantages to tumour cells ^86, 87^. However, it has been convincingly demonstrated that the total burden of mutations genome-wide in a tumour can represent the cumulative impact of mutagens, repair processes, and other influences along the path of tumourigenesis ^88, 89^. This overall burden of mutation can be disentangled to identify distinct and independent patterns of mutation which represent distinct mutational processes ^90, 91^. The associations seen between these patterns and particular DNA repair deficiencies were recently validated *in vitro* by using isogenic cell models ^92^. The computational analyses required to disentangle these patterns have been developed by using large pan-cancer cohorts of WGS/whole exome sequencing as well as for a large cohort of breast cancer WGS and have successfully identified over 30 patterns or signatures of SNVs classified by their substitution type and trinucleotide context ^93^. Analogous approaches have been proposed for the analyses of indels ^94^ and larger structural rearrangements ^20^; in the latter, SVs are classified on the basis of whether or not their breakpoints fall in a cluster of breakpoints in the genome. The rationale behind this separation is that clusters of breakpoints may be formed by mutational processes that are different from those that form dispersed breakpoints ^20, 89^. SVs are also classified by their type and size ^20^, and compound mutational signatures, including SNVs and SV breakpoints, can be constructed. This approach was successfully tested by using a large cohort of breast cancer WGS ^20^, resulting in the identification of characteristic signatures of rearrangement, reflecting HR deficiency, associated with the inactivating mutations of the *BRCA1/2* genes. This signature of HR deficiency could then be sought in other tumour samples lacking mutated *BRCA1/2* genes by using a new algorithm: HRDetect ^95^. This significantly expands the number of patients who have tumours showing HR deficiency (for example, due to epigenomic inactivation of *BRCA1/2* or mutations in other HR genes) and who are expected to respond to therapies exploiting defective repair, such as poly (ADP-ribose) polymerase (PARP) inhibitors ^95^. The authors also demonstrated the success of HRDetect in predicting HR deficiency in ovarian and pancreatic tumours, suggesting broader potential for therapeutic stratification of patients. Another study has since validated these results, showing that HRDetect provides clinically relevant information independently of *BRCA1/2* mutation status and that high HRDetect scores identified patients with good responses to platinum-based chemotherapy ^96^. However, the input to HRDetect relies on SV calls from a single SV caller. The incorporation of variant calls from additional callers to form consensus calls may be beneficial in ensuring the tool’s robustness. Beyond patient stratification, it has also been shown that SV mutational signatures can be used to predict overall survival in a cohort of HGSOC ^97^. Finally, it has been shown that the burden of SVs and SNVs in a tumour is associated with the degree of infiltration by host immune cells ^98^, linking the mutational state of a tumour to its tendency to elicit an immune response. At the cutting edge of cancer immunotherapy, efforts are under way to exploit such immunogenic tumour mutations and design individual anti-cancer vaccines, placing tumour genomics at the centre of oncology ^99^.

The last few decades have transformed our view of cancer to reveal each tumour as a largely unique constellation of SNV and SV mutations, and a tiny fraction of these mutations drive tumourigenesis ^100^. The complete reconstruction of the evolutionary trajectory travelled by a tumour could provide new biomarkers for rapid diagnosis and effective treatment and ultimately allow us to predict cancer progression. This comprehensive understanding is currently out of reach and, given the challenges involved, may remain so in the near future ^101^. However, in spite of our ignorance about the functional impacts of SVs and our modest success in detecting somatic mutations accurately, there are good reasons for optimism. Although we still lack the sample sizes required to detect all driver genes for most cancer types ^102^, there have already been unexpected benefits in amassing WGS tumour cohorts, with the genome-wide patterns of damage seen in a tumour providing novel biomarkers and guiding treatment. The next decade will see an explosion in the available data and improvements in analysis, making genomics a routine component of medical practice in oncology.

## Abbreviations

AID, activation-induced cytidine deaminase; CML, chronic myeloid leukaemia; CNA, copy number alteration; DSB, double-strand break; HGSOC, high-grade serous ovarian cancer; HR, homologous recombination; ICGC, International Cancer Genome Consortium; NHEJ, non-homologous end joining; RAG, recombination activating gene; SNV, single-nucleotide variant; SV, structural variant; TCGA, The Cancer Genome Atlas; WGS, whole genome sequencing

## Data and software availability

The data used to generate Figure 1 are available from the ICGC data portal at the following link: https://dcc.icgc.org/releases/current/Projects.
